# Pregnancy in a patient with metastatic functioning midgut neuroendocrine tumor receiving lanreotide after peptide receptor radionuclide therapy

**DOI:** 10.20945/2359-4292-2026-0102

**Published:** 2026-08-31

**Authors:** Beatriz Tavares da Silva, Ariana Maia, Maria Teresa Pereira, Sara Santos, Susana Garrido, Joana Vilaverde, Marta Sales Moreira, Clara Pinto, Ana Paula Santos, Jorge Dores

**Affiliations:** 1 Departamento de Endocrinologia, Centro Hospitalar Universitário de Santo António, Unidade Local de Saúde de Santo António, Porto, Portugal; 2 Departamento de Endocrinologia, Instituto Português de Oncologia Francisco Gentil – Núcleo do Porto/Porto Comprehensive Cancer (P.CCC), Porto, Portugal; 3 Departamento de Obstetrícia, Centro Hospitalar Universitário de Santo António, Unidade Local de Saúde de Santo António, Porto, Portugal; 4 Grupo de Abordagem de Lesões Pré-cancerosas e Cancro Precoce (PRECAM), Centro de Investigação do IPO Porto (CI-IPOP), Rede de Investigação em Saúde RISE@CI-IPO, Porto, Portugal

**Keywords:** Neuroendocrine tumors, pregnancy, lanreotide, carcinoid syndrome, peptide receptor radionuclide therapy, somatostatin analogs

## Abstract

Neuroendocrine tumors (NETs) are rare neoplasms that present significant challenges during pregnancy, particularly in the context of metastatic disease or carcinoid syndrome requiring systemic therapy. Data on the safety of somatostatin analogs during pregnancy remain limited and are primarily derived from studies involving patients with acromegaly. We report the maternal and fetal outcomes in a pregnant patient diagnosed with a metastatic NET of the ileocecal valve, complicated by carcinoid syndrome. The patient had previously undergone treatment with ^177^Lu-DOTATATE, debulking surgery, and lanreotide therapy. Lanreotide administration was continued throughout the pregnancy under multidisciplinary supervision. Maternal monitoring revealed no complications, and postpartum evaluations confirmed disease stability. The newborn developed transient neonatal hyperbilirubinemia requiring phototherapy but demonstrated normal development at the 18-month follow-up. This case highlights important clinical considerations regarding the continuation of somatostatin analog therapy, biochemical monitoring, peri-delivery planning for a carcinoid crisis, and pregnancy after peptide receptor radionuclide therapy in patients with functioning metastatic NETs. It provides supportive observational evidence that the continuation of lanreotide during pregnancy may be feasible in carefully selected patients under close multidisciplinary supervision, although conclusions regarding safety remain limited by the rarity of available data.

## INTRODUCTION

Neuroendocrine tumors (NETs) are a heterogeneous group of neoplasms that may arise in several organs, including the small intestine, pancreas, colon, and lungs (^1^,^2^). NETs may be benign or malignant, functional (hormone-secreting) or nonfunctional, as well as sporadic or associated with inherited syndromes (^2^).

Pregnancy in female patients undergoing systemic treatment for metastatic neuroendocrine neoplasms is rare, clinically challenging, and poorly documented, as these neoplasms are themselves rare. Consequently, clinical data on the maternal and fetal effects of treatment with somatostatin analogs (SSAs) in such patients are scarce (^3^). The limited available data on the safety of SSAs during pregnancy are primarily based on their use in acromegaly. Data regarding pregnancy after peptide receptor radionuclide therapy (PRRT) are even more limited.

Here, we describe the maternal and fetal outcomes of a pregnant woman previously diagnosed with a metastatic NET of the ileocecal valve accompanied by carcinoid syndrome, who had undergone treatment with ^177^Lu-DOTATATE and continued lanreotide therapy throughout her pregnancy under multidisciplinary supervision.

## CASE PRESENTATION

We report the case of a 33-year-old woman initially diagnosed with a metastatic grade 1 (G1) NET of the ileocecal valve and carcinoid syndrome at the age of 29. At the time of diagnosis, she presented with multiple liver and lymph node metastases, experiencing symptoms of abdominal pain, flushing, nausea, vomiting, diarrhea, and unintentional weight loss. Her medical history included multiple consultations with various specialists for allergies, a diagnosis of irritable bowel syndrome, and recurrent urinary tract infections. Baseline staging with ^68^Ga-DOTANOC PET/CT at the time of diagnosis demonstrated increased somatostatin receptor expression across multiple sites: two subcentimeter nodules in the left and right pericardial regions (interpreted as lymph node metastases), at least five hypermetabolic hepatic foci (notably, a 30-mm lesion at the segment IV/V junction), intense uptake in the body and tail of the pancreas, and focal uptake in the uncinate process. Additional uptake in the right lower quadrant suggested a small bowel NET with possible nodal involvement, and scattered mild-to-moderate uptake throughout the abdominopelvic cavity raised suspicion for peritoneal implants or synchronous disease.

Urinary 5-hydroxyindoleacetic acid (5-HIAA) and chromogranin A (CgA) levels were 61.08 mg/24 h (reference range [RR] 2.0–9.0) and 256.72 ng/mL (RR: < 98.1 ng/mL), respectively. A diagnosis of a G1 NET was confirmed through histopathological evaluation of a liver metastasis biopsy. The analysis demonstrated a well-differentiated NET with a Ki-67 index of 2% and a mitotic count of 2 per 2 mm^2^, showing concordant findings between the primary tumor and the liver metastases. Carcinoid heart disease was ruled out based on normal N-terminal pro-B-type natriuretic peptide (NT-proBNP) levels and a transthoracic echocardiogram, which revealed normal valvular morphology and function without evidence of right-sided heart involvement or chamber dilation.

Long-acting subcutaneous lanreotide was initiated at a dose of 120 mg every 28 days. Follow-up magnetic resonance imaging and ^68^Ga-DOTANOC PET/CT performed three months after starting lanreotide therapy demonstrated disease progression. There was increased radiotracer uptake in the ileocecal region (maximum standardized uptake value of 29.44), consistent with the known primary tumor, alongside new or more prominent uptake in previously identified sites: pericardial and epiphrenic lymph nodes, at least three hepatic metastases (with a maximum standardized uptake value of 72.51 in segment IV), pancreatic lesions, and abdominopelvic areas suggestive of further metastatic spread.

In light of this progression, ^177^Lu-DOTATATE therapy was proposed, and a total of four cycles were completed. PRRT was administered as a standard-of-care treatment rather than within a clinical trial protocol. Despite disease stabilization, cytoreductive (debulking) surgery was undertaken; this procedure consisted of an ileocolectomy, a cholecystectomy, a hepatic metastasectomy, and the excision of uterine fibroids. A total of three liver metastases were resected, and residual metastatic disease remained stable on subsequent imaging. The surgical approach aimed to reduce the tumor burden and improve long-term disease control, aligning with current practices for selected young, motivated patients with stable, low-grade (G1), well-differentiated NETs. Histological analysis confirmed a diagnosis of NET G1 (pT4N1M1) according to the European Neuroendocrine Tumor Society classification (^4^,^5^). Lanreotide treatment (120 mg every 28 days) was continued over the subsequent two years. During the follow-up, the patient showed clinical improvement and disease stability, achieving radiologic tumor volume reduction and the biochemical normalization of urinary 5-HIAA (3.0 mg/24 h) and CgA (39 ng/mL) levels.

Unexpectedly, three years after the initial diagnosis, the patient became pregnant while receiving long-acting subcutaneous lanreotide treatment. By the time she became aware of her pregnancy at 5 weeks of gestation, she had already received one dose of lanreotide. She was promptly referred to a specialized center dedicated to high-risk obstetrics to ensure appropriate maternal and fetal care. A chronological summary of the patient’s clinical course is presented in **Table 1**.

**Table 1.** Clinical timeline from diagnosis to postpartum follow-up

Age/timepoint	Clinical event
Age 29	Diagnosis of metastatic grade 1 (G1) ileocecal neuroendocrine tumor with carcinoid syndrome
At diagnosis	Elevated urinary 5-HIAA (61.08 mg/24h) and chromogranin A (256.72 ng/mL) ^68^Ga-DOTANOC PET/CT demonstrated liver, lymph node, pancreatic, and probable peritoneal metastatic involvement
Immediately after diagnosis	Lanreotide 120 mg every 28 days initiated
3 months later	MRI and ^68^Ga-DOTANOC PET/CT demonstrated disease progression
Age 29–30	Four cycles of 177Lu-DOTATATE administered
Following PRRT	Cytoreductive surgery performed (ileocolectomy, cholecystectomy, hepatic metastasectomy, and uterine fibroid excision)
Age 31 (following 2 years)	Stable disease under continuous lanreotide therapy with biochemical normalization
Age 33	Spontaneous pregnancy occurred while receiving lanreotide
5 weeks gestation	Pregnancy recognized after exposure to one dose of lanreotide
14 weeks gestation	First-trimester combined risk assessment performed
22 weeks gestation	Morphological fetal ultrasound showed no abnormalities
27 weeks gestation	Fetal echocardiography demonstrated normal findings
31 weeks gestation	Third-trimester ultrasound showed normal fetal growth (P48)
Third trimester	MRI demonstrated stable metastatic disease
39 weeks gestation	Elective induction of labor and vacuum-assisted vaginal delivery
Neonatal period	Transient neonatal hyperbilirubinemia requiring phototherapy
Age 34 (18 months postpartum)	Patient asymptomatic with stable disease; child with normal development

5-HIAA: 5-Hydroxyindoleacetic acid; G1: grade 1; MRI: magnetic resonance imaging; PET/CT: positron emission tomography/computed tomography; PRRT: peptide receptor radionuclide therapy.

After evaluation by a multidisciplinary team, a shared decision was made to continue lanreotide therapy. This decision considered the functioning and metastatic nature of the NET, as well as the limited clinical reports describing favorable maternal and fetal outcomes with SSA treatments, including lanreotide. A transthoracic echocardiogram and NT-proBNP measurement at this time revealed no abnormalities. The patient received subcutaneous lanreotide (120 mg every 28 days) throughout the entire pregnancy, with no dosage adjustments.

A first-trimester combined risk assessment was conducted at 14 weeks’ gestation, followed by a morphological ultrasound at 22 weeks and a fetal echocardiogram at 27 weeks. All evaluations showed normal fetal biometrics and no anomalies. A third-trimester ultrasound performed at 31 weeks revealed fetal growth at the 48^th^ percentile and no fetal abnormalities.

**Figure 1.**
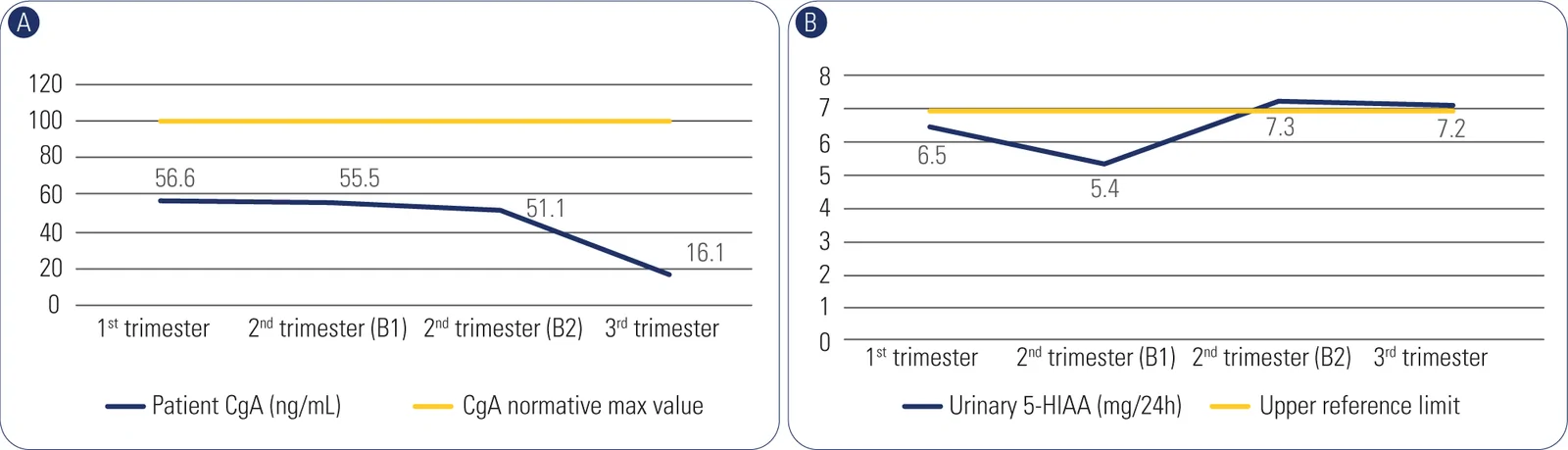
(**A**) Changes in chromogranin A (CgA) levels (reference range: 0–100 ng/mL) and (**B**) urinary 5-hydroxyindoleacetic acid (5-HIAA) levels (reference range: 1.0–7.0 mg/24 h) during pregnancy.

CgA: chromogranin A; 5-HIAA: 5-hydroxyindoleacetic acid; B1: first biochemical evaluation during the second trimester; B2: second biochemical evaluation during the second trimester.

Regarding maternal outcomes, standard screening protocols revealed no evidence of gestational hypertension, preeclampsia, or gestational diabetes. Biochemical analyses of CgA and urinary 5-HIAA levels remained within or near normal ranges throughout the pregnancy (**Figure 1**). A magnetic resonance imaging performed in the third trimester showed no evidence of metastatic disease progression. At 39 weeks’ gestation, the patient was admitted for an elective induction of labor. Epidural analgesia was administered. To shorten the expulsion stage, vacuum extraction and additional maneuvers were necessary to resolve fetal shoulder dystocia. The delivery proceeded without further complications, and a preplanned octreotide infusion protocol, intended for the prevention of a carcinoid crisis, was ultimately not required.

The male newborn presented with normal anatomical characteristics, a birth weight of 3,820 g, a length of 50.5 cm, and a head circumference of 37 cm — all parameters appropriate for gestational age. The Apgar scores at 1, 5, and 10 min were 8, 9, and 9, respectively, indicating good overall vitality. A pediatric evaluation within the first 24 h confirmed the absence of visible malformations or tremors and reported normal neonatal reflexes. Due to the lack of safety data regarding breastfeeding while on lanreotide, formula feeding was advised.

On his second day of life, jaundice was observed, and a transcutaneous bilirubin (TcB) level of 13.2 mg/dL (RR: 0.2–1.0 mg/dL) was recorded; this did not initially require phototherapy. However, by the fourth day, a clinical reassessment revealed a TcB level exceeding 20 mg/dL, requiring hospitalization for phototherapy. After 48 h of treatment, the newborn was discharged with a TcB level of 12.7 mg/dL. No congenital abnormalities or persistent developmental concerns were observed during the follow-up period. At two months of age, the infant exhibited normal psychomotor development. His somatometric parameters were as follows: a weight of 5510 g (50^th^ percentile), a length of 56 cm (15^th^ percentile), and a head circumference of 39 cm (50^th^ percentile). Eighteen months after delivery, the patient was still asymptomatic, maintaining normal levels of urinary 5-HIAA and serum CgA, and with stable disease on morphologic and functional imaging.

## DISCUSSION

Pregnancy in patients with metastatic midgut NETs and active carcinoid syndrome presents a complex therapeutic dilemma for which no formal guidance exists. We report a pregnancy with favorable maternal and fetal outcomes in a 33-year-old woman with a metastatic G1 ileocecal NET and carcinoid syndrome; conception occurred two years after the completion of PRRT, and the patient was managed with uninterrupted lanreotide (120 mg) throughout gestation. Despite the challenges of metastatic disease and the significant mechanical stress of a vacuum-assisted delivery complicated by shoulder dystocia, the patient maintained biochemical and radiological stability, resulting in the birth of a healthy, appropriate-for-gestational-age newborn. Although factors such as the psychological burden of the disease and the potential hormonal effects of systemic therapies may impair fertility in patients with NETs, pregnancy is increasingly encountered in clinical practice. This trend reflects both the prolonged survival achieved with modern SSAs and the rising age of first-time mothers, creating a need for clearer management strategies in this setting (^6^).

Safety of somatostatin analogs is the first-line systemic therapy for metastatic G1-G2 NETs and are a key component of carcinoid syndrome management (^7^,^8^). Although SSAs are known to cross the placental barrier via passive diffusion, *in vitro* studies suggest they have low-affinity binding to placental somatostatin receptors (^9^). Importantly, most previously reported cases of pregnancy in patients with metastatic NETs treated with lanreotide describe either the interruption of therapy during the first trimester or its reintroduction later in gestation (^10^–^12^). Published cases of pregnancy in patients with metastatic NETs receiving lanreotide are summarized in **Table 2**. In contrast, our patient received uninterrupted lanreotide therapy from early pregnancy until delivery. This decision was not based solely on theoretical safety, but on the clinical need to preserve hormonal stability in a patient with active carcinoid syndrome and a significant metastatic burden. The stable urinary 5-HIAA and CgA levels observed throughout pregnancy, combined with the absence of radiological progression, suggest that maintaining antisecretory therapy may be feasible in selected patients with functioning metastatic NETs under close multidisciplinary supervision.

**Table 2.** Published cases of pregnancy in patients with metastatic neuroendocrine tumors receiving lanreotide

Reference	Age at pregnancy (years)	Site of primary tumor	Grade	Treatment during pregnancy	Treatment before pregnancy	Pregnancy outcomes	NET status postpartum
Giulia Meoni et al. (^10^)	41	Ileal NET (breast, axillary lymph nodes, and liver metastases)	G2	Lanreotide depot (LAR) 120 mg every 28 days	LAR 120 mg every 28 days	Cesarean delivery due to hypertensive emergency (history of chronic hypertension before pregnancy). No other adverse maternal and fetal outcomes.	Stable disease
	37	Pancreatic NET (liver and bone metastases)	G2	LAR 120 mg every 28 days	Distal splenopancreatectomy and liver metastasis resection. LAR 120 mg every 28 days	Cesarean delivery. No adverse maternal and fetal outcomes.	Disease progression (1 year postpartum; everolimus added with disease stabilization)
Nynne Emilie Hummelshøj et al. (^11^)	32	Small intestinal NET (liver metastasis)	G1	1^st^ trimester: lanreotide discontinuation 16 weeks: lanreotide resumed with dose titration to 120 mg every 28 days	LAR 120 mg every 28 days, debulking surgery	Cesarean delivery. No adverse maternal and fetal outcomes.	Stable disease
Ratnayake et al. (^12^)	35	Midgut NET (liver and pancreas metastases)	G2	Octreotide, LAR	Surgery, octreotide, LAR	Emergency cesarean delivery at 31 weeks (preterm pre labor rupture of membranes). Invasive ventilation at neonatal intensive care, nasogastric tube feeding, retinopathy of prematurity, ventriculomegaly, mild developmental delay in the baby	Stable disease
	31	Pancreatic (liver, abdominal node, pancreas metastases)	G2	Octreotide, LAR	Octreotide, LAR	Cesarean delivery. No adverse maternal and fetal outcomes.	Disease progression
	33	Midgut (liver, midgut, portocaval, pelvic and retroperitoneal metastases)	G2	Octreotide, LAR (not in 1^st^ trimester)	Octreotide, LAR	Cesarean delivery. No adverse maternal and fetal outcomes.	Stable disease
	36	Midgut (liver, midgut, portocaval, pelvic and retroperitoneal metastases)	G2	Octreotide, LAR	Octreotide, LAR	Cesarean delivery. No adverse maternal and fetal outcomes.	Not available
	33	Pancreatic (liver metastases)	G2	LAR	LAR, PRRT within a year before pregnancy	Cesarean delivery. Gestational diabetes controlled by diet.	Stable disease

LAR: Long-acting formulation; NET: neuroendocrine tumor; PRRT: peptide receptor radionuclide therapy.

The safety profile observed in our patient aligns with data from populations with acromegaly, in which SSAs are not consistently associated with adverse maternal and fetal outcomes (^13^). However, the clinical context differs substantially. In acromegaly, SSAs are often discontinued unless significant tumor growth occurs, whereas in metastatic NETs, the risk of acute clinical deterioration due to uncontrolled carcinoid syndrome is considerably higher. Particularly in midgut NETs with hepatic metastases, serotonin-mediated complications and the increased hemodynamic workload of pregnancy necessitate a more cautious approach to treatment interruption (^8^,^9^,^14^). This case highlights the importance of individualized risk-benefit assessments when considering the continuation or interruption of SSA therapy during pregnancy.

An additional important aspect of this case was the interpretation of biochemical markers during pregnancy. A mild increase in urinary 5-HIAA was observed from the second trimester onward. Rather than definitively indicating tumor activity, this finding may partially reflect physiological, pregnancy-related changes, including increased placental serotonin production and plasma volume expansion (^15^). Recognizing this physiological interference is crucial to avoid misinterpretation and unnecessary therapeutic adjustments in patients with functioning NETs during pregnancy.

Our case also underscores the importance of anticipating the risk of a carcinoid crisis during delivery. Functioning NETs carry a well-recognized risk of hemodynamic instability during anesthesia or high-stress events due to massive amine release (^16^). Therefore, a preventive octreotide infusion protocol was prepared in advance. Notably, despite the need for vacuum extraction and additional maneuvers for shoulder dystocia, both of which represent significant physical stressors, no carcinoid crisis occurred. Continuous lanreotide therapy throughout the third trimester may have contributed to this biochemical stability, although this remains speculative (^17^). Such anticipatory peripartum planning represents a critical, yet rarely described, component of management in pregnant patients with carcinoid syndrome.

A particularly distinctive feature of this report is the successful pregnancy following prior PRRT. Data regarding pregnancy outcomes after 177Lu-DOTATATE therapy are extremely limited, although successful pregnancies following PRRT have been previously reported (^18^,^19^). Concerns also persist regarding potential adverse effects on the pituitary-gonadal axis due to somatostatin receptor expression (^20^). While some studies have reported transient changes in gonadotropin levels, recent data have not demonstrated a clear association with clinically significant hypogonadism or subfertility in premenopausal women (^21^–^23^). The ability of our patient to conceive spontaneously and maintain a stable G1-pT4N1M1 NET two years after PRRT adds meaningful, real-world reassurance in an area where evidence remains scarce.

Compared with previously published cases of pregnancy in patients with metastatic NETs receiving lanreotide, to our knowledge, this report uniquely combines prior PRRT, active carcinoid syndrome, uninterrupted SSA therapy throughout gestation, and structured peripartum planning for a carcinoid crisis (^10^–^12^). These elements provide practical information that extends beyond the demonstration of drug safety and contribute directly to clinical decision-making.

Despite the favorable outcome, this report has limitations, including its single-case nature and the absence of long-term pediatric follow-up. Nevertheless, in rare clinical scenarios where prospective studies are neither ethical nor feasible, well-documented, real-world cases play an essential role in guiding multidisciplinary management.

In conclusion, this case suggests that a pregnancy with favorable maternal and fetal outcomes is possible in selected patients with metastatic functioning NETs following PRRT and during continuous lanreotide therapy. Careful multidisciplinary planning, maintenance of hormonal stability, and cautious interpretation of biochemical markers are important considerations in selected patients managed during pregnancy.

## Data Availability

datasets related to this article will be available upon request to the corresponding author.
